# Supplementary material for: Structure and activity of the essential UCH family deubiquitinase DUB16 from Leishmania donovani
Source: Biochem J. 2025 Jul 9;482(14):969–88. doi: 10.1042/BCJ20253107 (PMC12409989; doi:10.1042/BCJ20253107)
Supplement: Online supplementary table 1 [file bcj-482-14-BCJ20253107-s001.pdf]

## **Supplementary Table S1**.

| | LdDUB16 | LdDUB15 | HsUCHL1 | HsUCHL3 | HsUCHL5 | HsBAP1 |
|---|---|---|---|---|---|---|
| **LdDUB16** | - | **26** | **33** | **37** | **25** | **26** |
| **LdDUB15** | | **-** | **24** | **25** | **41** | **34** |
| **HsUCHL1** | | | **-** | **55** | **23** | **24** |
| **HsUCHL3** | | | | **-** | **29** | **24** |
| **HsUCHL5** | | | | | **-** | **47** |
| **HsBAP1** | | | | | | **-** |

Sequence identities among the UCH type DUBs of *L. donovani* and the human host. The sequences used in the alignment were LdDUB16 (UNIPROT Accession A0A504WVB8) residues 1-233; LdDUB15 (A0A3S7WY30) residues 1-230; HsUCHL1 (P09936) residues 1-223; HsUCHL3 (P15374) residues 1-230; HsUCHL5 (Q9Y5K5) residues 1-228; HsBAP1 (Q92560) residues 1-240.

## Supplementary Table S2. Oligonucleotide primers, coding sequences and recombinant proteins

| | |
|---|---|
| DUB16_F | 5′ ACTGTGGATCC*ATGTGGTTCCCACTGGAGAGCAATC* 3′ |
| DUB16_R | 5′ GGTCAGAAGCTTCTATTA*TCACTTGTTCACGAGTGCGGTGATG* 3′ |
| DUB16For | 5′ GTGGCCTCATATG*TGGTTCCCACTGGAGAGCAATC* 3′ |
| DUB16Rev | 5′ CGGCAAGCTTA*TCACTTGTTCACGAGTGCGGTGAT* 3′ |
| UbL40 _F | 5′ CCATGGCG*ATGCAGATCTTCGTGAAGACGC* 3′ |
| UbL40 _R | 5′ CTCGAGTCTTAA*CTTCTTCTTCATGCGCAGGTTGG* 3′ |
| LdBPK_250190.1 coding sequence | ATGTGGTTCCCACTGGAGAGCAATCCGCAGGTGATGAACCGCTACATCAGCACCTTGGG<br>TCTCACAGAAGCGAAGGTGCAGTTTGTGGACGTGTACGGTGTGTCGGACGATCTACTTG<br>AAATGGTGCCTTCGCCGGTGCATGCCGTGCTCCTCGTGTACCCCATGTGCGAGGCCACG<br>GATAGACGCCTAGCGGAGCAGCAGGCTGCACAGACAGCGGAAGTTGCGGCGCTTCGCA<br>AATCACACCCTTTCTTCTTCACACACCAGCTCGTCCCGAACGCGTGCGGAACCATCGCCAT<br>CGCGCATGCTCTTATGAACAACCGCGATAAGCTCGGTGAGATCGCCGCCGGCAGCATCC<br>TCGACGGCCCGTGGGCGAAGGCGGCAGAAACGTCGGAAGATCCCCAGATCATCGGAAA<br>ACTTATTGCGGAAGACACGAGTCTCGCCAGCGCTCACGCCGCTGCTGCGCAGGAGGGCG<br>CGACCGCCAACCAACACATCGACGCGGACATCGACCTTCACTTTGTCTGCTTCATCCCCGT<br>AGGCGGACGCTGCGTTGAGCTGGATGGGCGAAAAGAGAACCCGATCTTGCACGGTACC<br>TGCACCGACAACAGGTCTTTCCTCACCGCTGCAGCCGCCGCGATACAGGAACGCGTAGA<br>GCTCAACCCCAGCTCCTACGAGTTTGGCATCACCGCACTCGTGAACAAGTGA |
| Recombinant LdDUB16 protein sequence | **MGSSHHHHHHSSGLEVLFQGPA**MWFPLESNPQVMNRYISTLGLTEAKVQFVDVYGVSDD<br>LLEMVPSPVHAVLLVYPMCEATDRRLAEQQAAQTAEVAALRKSHPFFFTHQLVPNACGTIAI<br>AHALMNNRDKLGEIAAGSILDGPWAKAAETSEDPQIIGKLIAEDTSLASAHAAAAQEGATAN<br>QHIDADIDLHFVCFIPVGGRCVELDGRKENPILHGTCTDNRSFLTAAAAAIQERVELNPSSYEF<br>GITALVNK |
| LdBPK_311930 coding sequence | ATGCAGATCTTCGTGAAGACGCTGACCGGCAAGACGATCGCGCTGGAGGTGGAGCCGA<br>GCGACACGATCGAGAACGTGAAGGCGAAGATCCAGGACAAGGAGGGCATCCCGCCGG<br>ACCAGCAGCGCCTGATCTTCGCCGGCAAGCAGCTGGAGGAGGGCCGCACGCTCTCGGA<br>CTACAACATCCAGAAGGAGTCCACGCTGCACCTGGTGCTGCGCCTGCGCGGCGGCGTGA<br>TGGAGCCGACGCTGGTCGCGCTGGCCAAGAAGTACAACTGGGAGAAGAAGGTGTGCCG<br>CCGCTGCTACGCCCGCCTGCCGGTGCGCGCCACGAACTGCCGCAAGAAGGCCTGCGGTC<br>ACTGCTCCAACCTGCGCATGAAGAAGAAGCTGCGCTAG |
| Recombinant Ld Ub-L40 protein sequence | **MA**MQIFVKTLTGKTIALEVEPSDTIENVKAKIQDKEGIPPDQQRLIFAGKQLEEGRTLSDYNIQ<br>KESTLHLVLRLRGGVMEPTLVALAKKYNWEKKVCRRCYARLPVRATNCRKKACGHCSNLRM<br>KKKLR**LEHHHHHH** |

**Supplementary Table S3 Dose-Response Results**

| | M379 | DUB16A | DUB16B |
|---|---|---|---|
| log(inhibitor)vs. normalized response-- Variable slope | | | |
| Best-fit values | | | |
| $LogIC_{50}$ | 0.3373 | 0.5624 | 0.5068 |
| HillSlope | -3.873 | -4.432 | -3.564 |
| $IC_{50}$ | 2.174 | 3.651 | 3.212 |
| 95% CI (profile likelihood) | | | |
| $LogIC_{50}$ | 0.3083 to 0.3679 | 0.5378 to 0.5847 | 0.4687 to 0.5428 |
| Hill Slope | -5.192 to -3.097 | -9.972 to -3.383 | -4.871 to -2.791 |
| $IC_{50}$ | 2.034 to 2.333 | 3.450 to 3.844 | 2.942 to 3.490 |
| Goodness of Fit | | | |
| Degrees of Freedom | 79 | 79 | 79 |
| R squared | 0.9516 | 0.9613 | 0.9321 |
| Sum of Squares | 7666 | 6142 | 10686 |
| Sy.x | 9.851 | 8.817 | 11.63 |
| | | | |
| Number of points | | | |
| # of X values | 81 | 81 | 81 |
| # Y values analyzed | 81 | 81 | 81 |
