# Supplementary material for: Structure and activity of the essential UCH family deubiquitinase DUB16 from Leishmania donovani
Source: Biochem J. 2025 Jul 9;482(14):969–88. doi: 10.1042/BCJ20253107 (PMC12409989; doi:10.1042/BCJ20253107)
Supplement: Online supplementary figure 1 [file bcj-482-14-BCJ20253107-s002.pdf]

Supplementary Figure S1

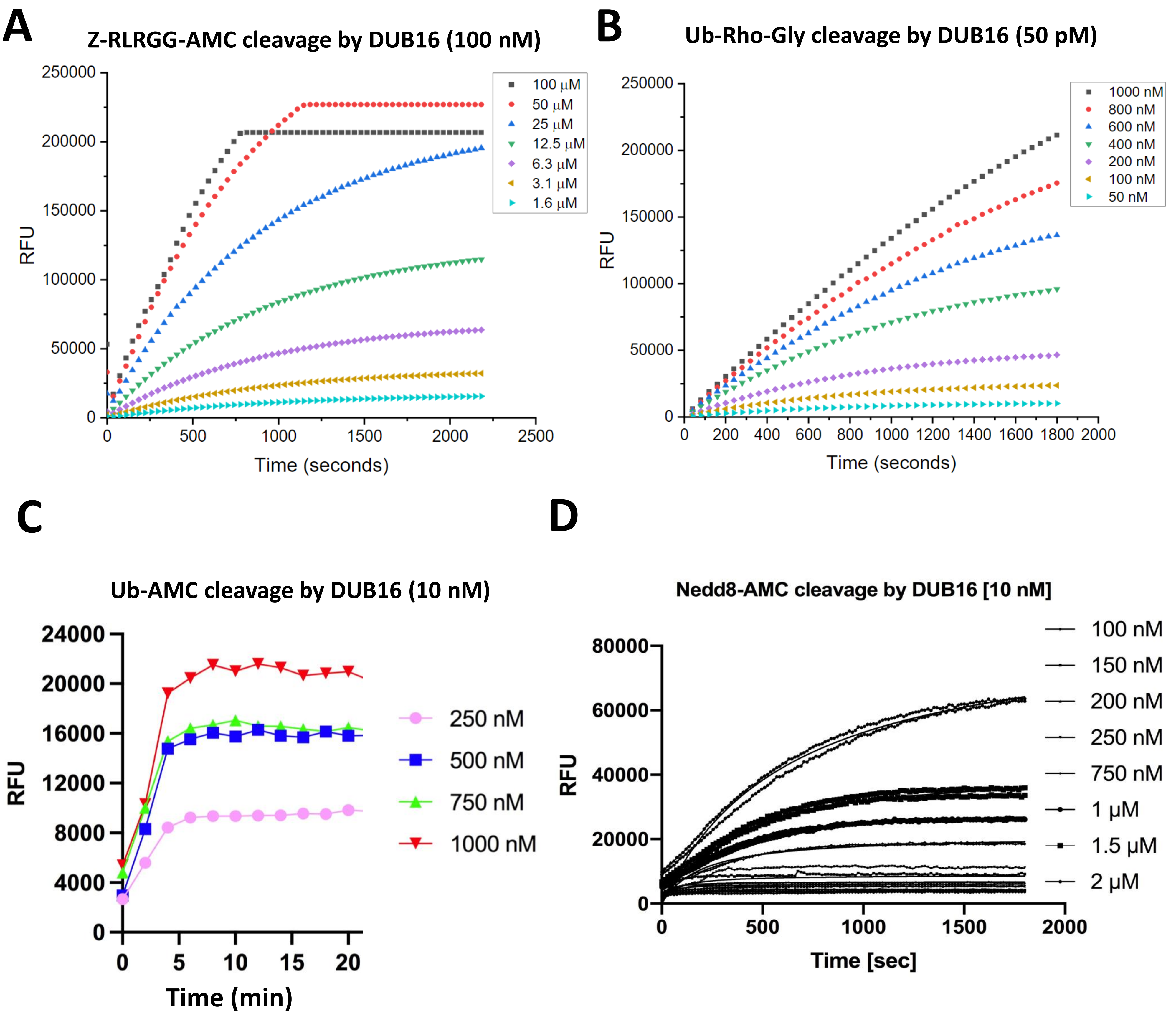

Time course of LdDUB16 cleavage of Peptide, Ub and Nedd8 small molecule conjugates. Cleavage of A) Z-RLRGG-AMC B) Ub-Rho-Gly C) Ub-AMC and D) Nedd8-AMC. Enzyme was incubated with substrate at the concentrations indicated in the keys and product formation is measured in relative fluorescence units (RFU).
