# Supplementary material for: Structure and activity of the essential UCH family deubiquitinase DUB16 from Leishmania donovani
Source: Biochem J. 2025 Jul 9;482(14):969–88. doi: 10.1042/BCJ20253107 (PMC12409989; doi:10.1042/BCJ20253107)
Supplement: Online supplementary figure 2 [file bcj-482-14-BCJ20253107-s003.pdf]

# Supplementary Figure S2

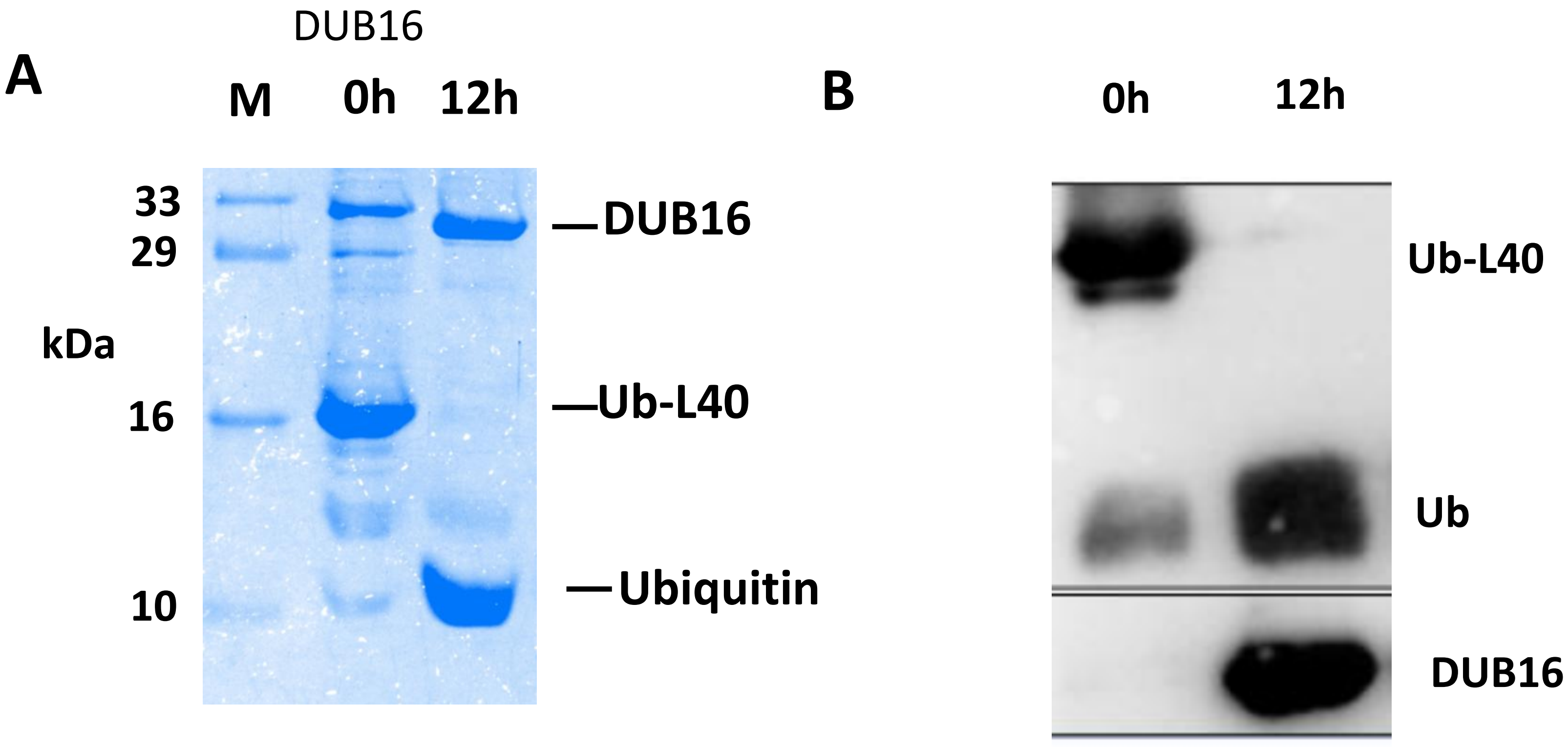


Cleavage of the Ub-L40 fusion protein by LdDUB16 – reactions run to completion overnight . **A**. Products were visualised by Coomassie staining following SDS-PAGE. The L40 product is less stable and is not seen here. **B**. Visualisation following immunoblotting with ubiquitin specific antibodies (Upper) These blots were reprobed with anti-DUB16 antibody (Lower).
