# Supplementary material for: Structure and activity of the essential UCH family deubiquitinase DUB16 from Leishmania donovani
Source: Biochem J. 2025 Jul 9;482(14):969–88. doi: 10.1042/BCJ20253107 (PMC12409989; doi:10.1042/BCJ20253107)
Supplement: Online supplementary figure 3 [file bcj-482-14-BCJ20253107-s004.pdf]

Supplementary Figure S3

A

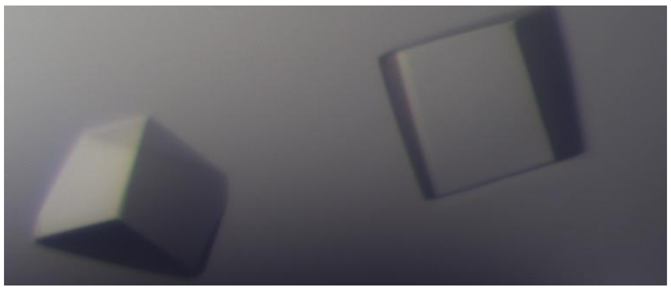

Crystals of LdDUB16 grown in sitting drops composed of equal volumes of protein (16 mg/ml in 150 mM NaCl, 25 mM Tris-HCl pH 8.0, 1 mM DTT) and a crystallisation solution of 1.9 M ammonium sulphate in 0.1 M Bis-Tris propane buffer (pH 7.0).

B

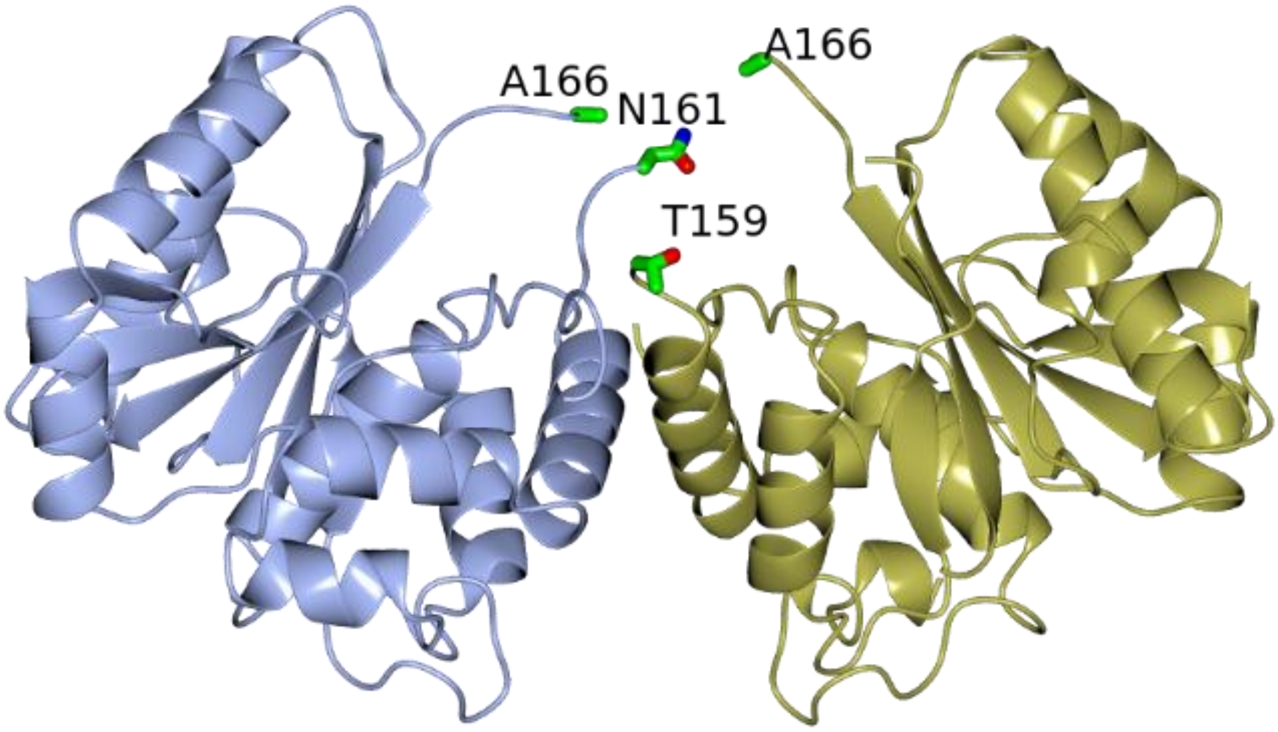

Ribbon rendering of the A and B molecules of the LdDUB16 asymmetric unit coloured blue and gold respectively. The Cα and side chains of esidues flanking the disordered residues of the crossover loop are displayed emphasing the close approach and the possibility of domain swapping.
