# Supplementary material for: Structure and activity of the essential UCH family deubiquitinase DUB16 from Leishmania donovani
Source: Biochem J. 2025 Jul 9;482(14):969–88. doi: 10.1042/BCJ20253107 (PMC12409989; doi:10.1042/BCJ20253107)
Supplement: Online supplementary figure 4 [file bcj-482-14-BCJ20253107-s005.pdf]

Supplementary Figure S4

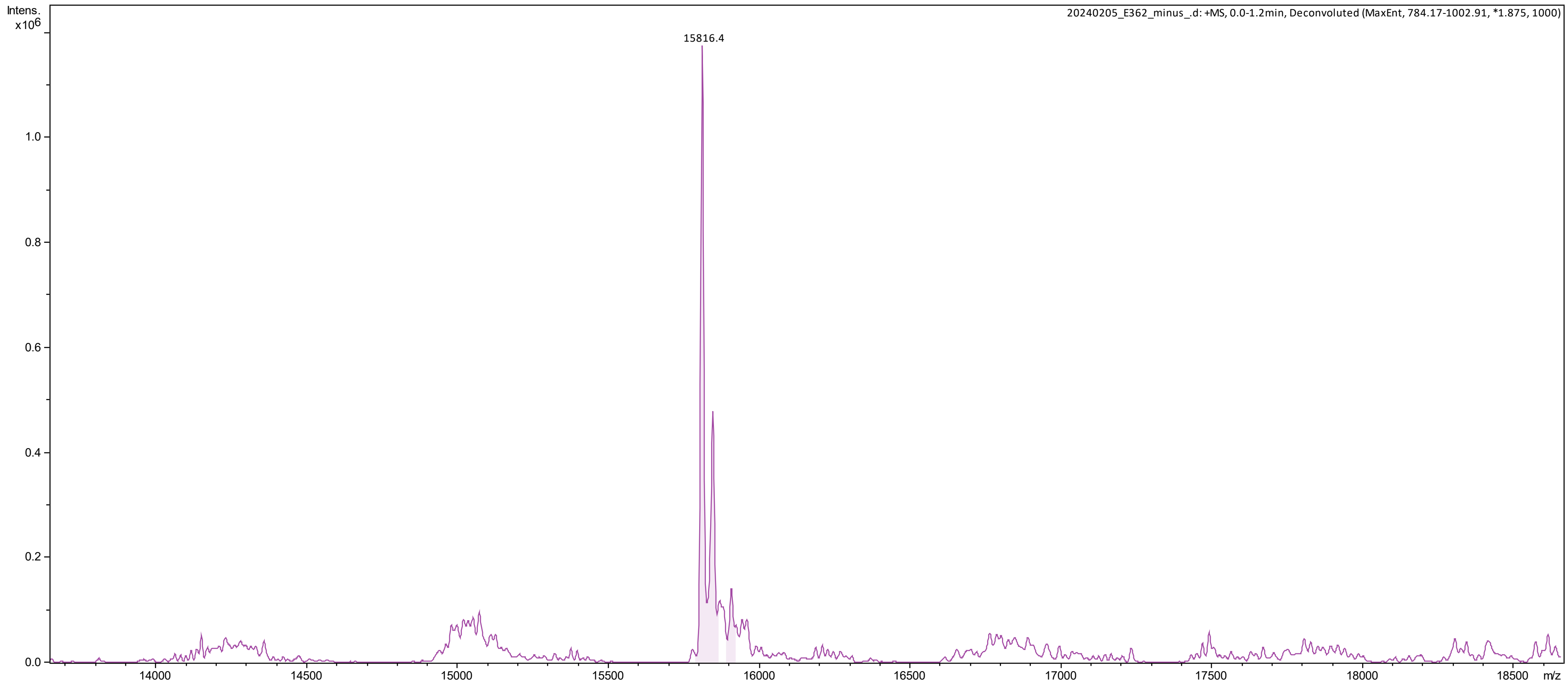

Analysis of the Ub-L40 sample diluted in aqueous 50% acetonitrile containing 1% formic acid by ESI-MS using direct infusion at 3 μl/min and a Bruker maXis qTOF mass spectrometer. Acquired Mass spectra were summed for each spectrum and deconvoluted to neutral average mases using MaxEnt with a resolution setting of 1K-5K. The mass calculated from the sequence of recombinant Ub-L40 is 15949.6 Da. The experimental mass of 15816.4 Da is consistent with loss of the N-terminal Met1 and the presence of an additional –S-S- bond – theoretical mass of 15816.4 Da

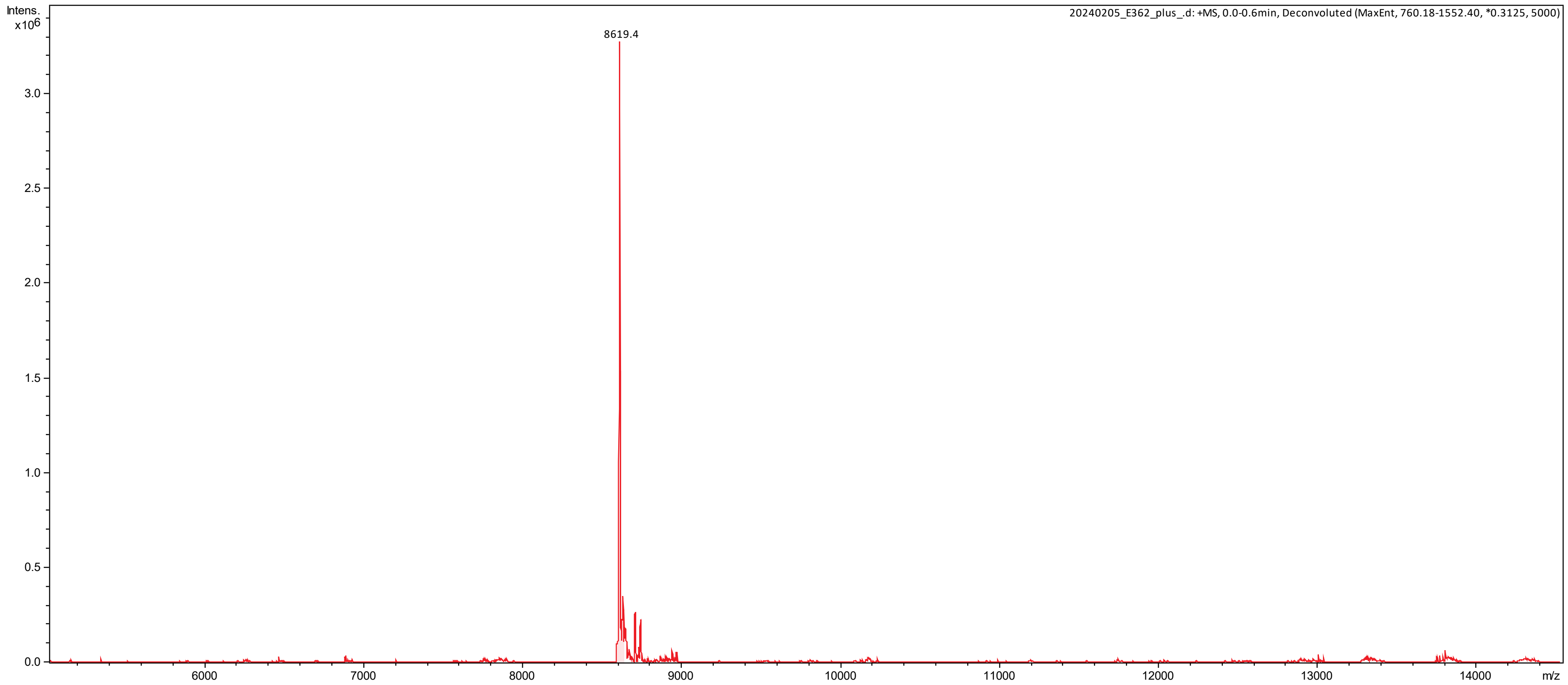

Analysis of the Ub-L40 following treatment with LdDUB16. Sample prepared and analysed as above. The experimentally measured mass of 8619.4 is an exact match to that of ubiquitin with an additional N-terminal alanine – a vestige of the cloning steps. This shows that cleavage has taken after the C-terminal RGG of ubiquitin.
